# Directional and monochromatic thermal emitter from epsilon-near-zero conditions in semiconductor hyperbolic metamaterials

**DOI:** 10.1038/srep34746

**Published:** 2016-10-05

**Authors:** Salvatore Campione, Francois Marquier, Jean-Paul Hugonin, A. Robert Ellis, John F. Klem, Michael B. Sinclair, Ting S. Luk

**Affiliations:** 1 Sandia National Laboratories, P.O. Box 5800 Albuquerque NM 87185 USA; 2 Center for Integrated Nanotechnologies (CINT), Sandia National Laboratories, P.O. Box 5800 Albuquerque NM 87185 USA; 3 Laboratoire Charles Fabry, Institut d Optique, CNRS–Univ Paris-Sud, Campus Polytechnique, RD128, 91127 Palaiseau Cedex, France

## Abstract

The development of novel thermal sources that control the emission spectrum and the angular emission pattern is of fundamental importance. In this paper, we investigate the thermal emission properties of semiconductor hyperbolic metamaterials (SHMs). Our structure does not require the use of any periodic corrugation to provide monochromatic and directional emission properties. We show that these properties arise because of epsilon-near-zero conditions in SHMs. The thermal emission is dominated by the epsilon-near-zero effect in the doped quantum wells composing the SHM. Furthermore, different properties are observed for *s* and *p* polarizations, following the characteristics of the strong anisotropy of hyperbolic metamaterials.

The mid-infrared region of the electromagnetic spectrum (2.5–25 μm) is highly interesting in many applications such as spectroscopy^1^, sensing^2^, and thermography^3^. In this frequency range, only a few kinds of sources are available, mainly quantum cascade lasers and thermal sources (blackbodies). The latter can be inexpensive; but suffer from poor efficiencies which can be as low as 10^−4^ for spectroscopic applications^4^. Because convection losses can be suppressed by operating under vacuum and conduction losses can be suppressed by a proper design^5^, the ultimate efficiency limit for incandescent sources appears to be due to emission into unwanted frequencies and directions. The development of novel sources that control the emission spectrum and the angular emission pattern is thus of fundamental importance. In the last few years, research on thermal radiation has led to the achievement of 1) *spatially coherent* (*i.e. directional*)^6^,^7^,^8^,^9^,^10^ or 2) *temporally coherent* (*i.e. narrow-band*)^11^,^12^,^13^,^14^ thermal sources by using wavelength-scale optical structures^6^,^13^,^15^,^16^,^17^. A few recent papers even succeeded in combining both properties of directionality and monochromaticity using diffraction order engineering in periodic structures^4^,^18^. It is also desirable that thermal radiation sources be capable of rapid modulation. Structures displaying a so-called epsilon-near-zero (ENZ) mode^19^,^20^,^21^ have been studied as potential thermal sources. Thermal-radiation control has been demonstrated from such devices^22^ and electrical control of the reflectivity^20^ and emissivity^17^ have been shown. Very recently, high-speed modulation of thermal emission has been demonstrated using a quantum well stack and a photonic crystal^23^.

In this work, we will show that simple multilayer structures without any periodic corrugation have the potential to behave as *directive and monochromatic* thermal sources in the infrared. To do so, we take advantage of an absorption resonance in a semiconductor hyperbolic metamaterial (SHM). This kind of structure is possible at mid-infrared frequencies since highly doped semiconductor materials behave like metals (i.e. have a large negative real part of the permittivity) so that SHMs can be fabricated using alternating layers of doped and undoped semiconductor materials^24^,^25^,^26^,^27^,^28^. Recent work has shown that the thermal radiation properties of layered metal/dielectric hyperbolic metamaterials are nearly flat and featureless in the mid-infrared part of the spectrum, and are not much different from those of simpler metallic structures^29^. However, in this work, we will show that this is not the case when the thermal radiation is generated in proximity of the ENZ frequency of the quantum wells composing the SHM. To be more precise, the resonance frequency and directivity are driven by an ENZ effect and the optical anisotropy of the hyperbolic metamaterial.

At given temperature *T*, wavelength *λ*, and direction *θ*, the thermal radiation intensity emitted from a body is


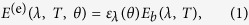


where *ε*_*λ*_(*θ*) is the emissivity of the device at wavelength *λ* and direction *θ*, and *E*_*b*_(*λ*, *T*) is the intensity of blackbody radiation at wavelength *λ* and temperature *T* determined by Planck’s law. The emissivity thus behaves as a “filter” of the blackbody spectral radiance and characterizes the way a given body will emit thermal radiation. The polarization dependence is also included in the emissivity. Kirchhoff’s law states that *α*_*λ*_(*θ*) = *ε*_*λ*_(*θ*)^30^ where *α*_*λ*_(*θ*) is the absorptivity at wavelength *λ* and direction *θ*. We will make use of this relationship to confirm the spectral and angular properties of thermal radiation of our samples.

## Results

**Figure 1:**
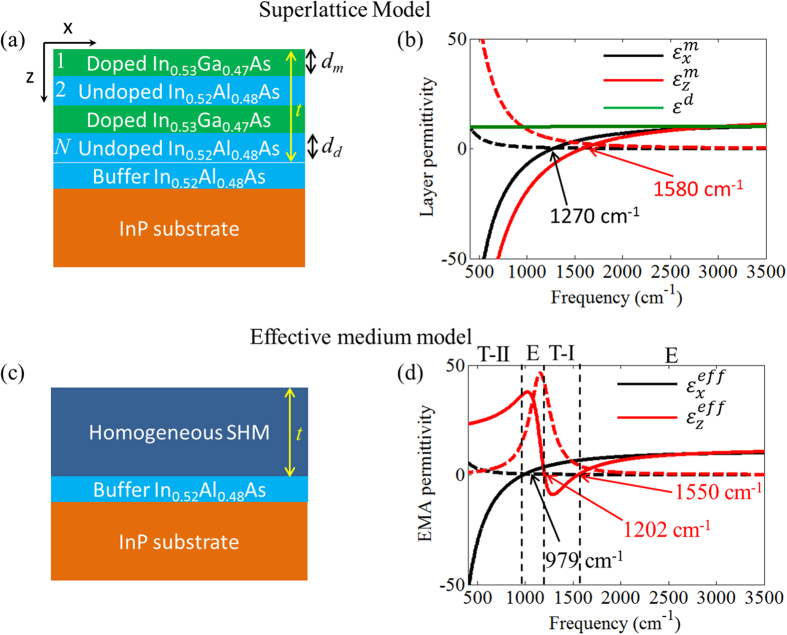
(**a**) The SHM is made by alternating 50 pairs of 10-nm-thick doped In_0.53_Ga_0.47_As and 8-nm-thick undoped Al_0.48_In_0.52_As layers. The superlattice is on top of a 200-nm-thick buffer Al_0.48_In_0.52_As layer and a 0.65-mm-thick InP substrate. (**b**) Permittivity functions of the layers composing the SHM extracted via ellipsometry. (**c**) The superlattice in (**a**) can be modeled as a homogeneous uniaxial SHM by using the effective medium model. (**d**) Effective permittivity functions of the homogeneous SHM computed using a local anisotropic effective medium model through the optical constants in (**b**). In panels (**b**,**d**), solid and dashed lines indicate real and imaginary parts of the permittivity, respectively. The vertical dashed lines in (**d**) separate frequency regions with a type-II hyperbolic dispersion (T-II), an elliptic dispersion (E), and a type-I hyperbolic dispersion (T-I) region.

The sample was grown using molecular beam epitaxy on a 0.65-mm-thick InP substrate with a 200 nm thick In_0.52_A_l0.48_As buffer layer. As shown in Fig. 1(a), the sample comprises 50 periods of alternating layers of 10 nm thick In_0.53_Ga_0.47_As quantum wells and 8 nm thick In_0.52_Al_0.48_As barriers. The In_0.53_Ga_0.47_As quantum wells are highly doped (2 × 10^19^ cm^−3^) and behave as a metal at low frequencies, while the barriers are undoped and behave as a dielectric at all frequencies. The sample was characterized using infrared variable angle spectroscopic ellipsometry (IR-VASE, J.A. Woollam Co.), as described in the Methods section. Ellipsometry measurements were obtained at five different incidence angles, and spanned the spectral range from 400 to 3500 cm^−1^. These measurements revealed that the doped In_0.53_Ga_0.47_As layer should be described as a *uniaxial Drude material*, with different Drude model parameters for the in-plane (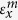) and out-of-plane (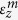) permittivities, where the superscript *m* indicates that the doped layer acts as the metallic layer. [Note that although we are using the *x* component to describe the in-plane permittivity, the permittivity is isotropic in the *x*-*y* plane]. The uniaxial behavior arises due to the electron confinement within the narrow (10 nm) quantum wells, which in turn leads to a blue-shifting of the plasma frequency of 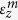 relative to that of 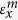 (see Fig. 1(b)) as described in refs 31 and 32. The ENZ point of the in-plane permittivity (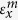) occurs at ~1270 cm^−1^ while that of the out-of-plane permittivity (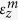) occurs at ~1580 cm^−1^.

In what follows, we will describe the behavior of the SHM sample using two different models. The first model, which we call the *superlattice model*, calculates the electromagnetic response of the SHM using the transfer matrix method^33^ explicitly considering all the layers of the SHM structure. The second model, which we call the *effective medium model*, uses the measured permittivities of the quantum well and barrier layers, along with the local anisotropic effective medium approximation^34^, (see the Methods section) to describe the SHM as a single uniaxial slab with an in-plane permittivity 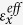 and an out-of-plane permittivity 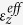
(see Fig. 1(c,d)). The thickness of the effective medium slab is equivalent to the total thickness of the 50 periods of the superlattice. In the effective medium model, the electromagnetic properties of the sample are once again calculated using the transfer matrix method, which now substitutes a single uniaxial slab for the 50 period quantum well structure. As we shall see, both models successfully recover the spectral and angular properties of the thermal radiation of the SHM structure. However, we find that a deeper understanding of the physical origin of the thermal radiation features, which are due to the occurrence of an epsilon-near-zero condition in the doped quantum wells, is only obtained through use of the superlattice model.

The permittivities obtained using the effective medium model are shown in Fig. 1(d), and exhibit the usual anisotropic behavior of hyperbolic metamaterials, where 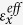 has a Drude-like behavior and 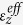 a Lorentz-like behavior. In the present case, the SHM exhibits type-II hyperbolic dispersion below ~979 cm^−1^, and type-I hyperbolic dispersion between ~1200 cm^−1^ and ~1550 cm^−1^. The sample exhibits elliptic dispersion in other frequency ranges. In particular, we note that 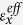
experiences an epsilon-near-zero condition at ~979 cm^−1^, while the ENZ condition of 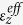 occurs at ~1550 cm^−1^.

**Figure 2:**
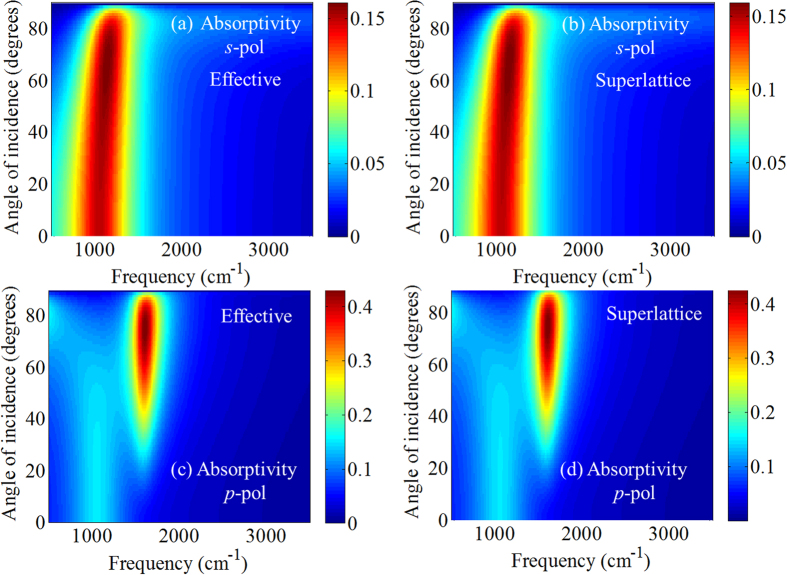
Absorptivity versus frequency and incidence angle under (**a,b**) *s*-pol and (**c,d**) *p*-pol plane wave incidence for SHMs as in Fig. 1. Results computed using (**a,c**) the effective medium approximation and (**b,d**) the superlattice implementation.

The sample’s polarized absorptivity (and hence emissivity) was calculated as 1−*R*−*T*, where *R* and *T* are the simulated reflectivity and transmissivity, respectively (see the Methods section). Figure 2 shows the *s*-polarized and *p*-polarized absorptivity versus frequency and incidence angle, calculated using both the superlattice and effective medium models—their agreement is strikingly evident. For *s*-polarized incidence, a single absorption feature is observed near ~1060 cm^−1^. For *p*-polarized incidence, two features are present at larger angles: a weak feature near ~1060 cm^−1^, and a much stronger feature near ~1600 cm^−1^. The weak feature near 1060 cm^−1^ originates from a slab impedance matching condition^24^ (i.e. Fabry-Perot resonance) and is similar in magnitude to the ~1060 cm^−1^ feature observed in *s*-polarization. As will be discussed below, the strong *p*-polarized peak observed near ~1600 cm^−1^ is associated with an *epsilon-near-zero condition of the doped quantum wells*. Thus, knowledge of the optical characteristics of the component layers of the SHM stack is necessary for a full physical understanding of the observed optical properties.

**Figure 3:**
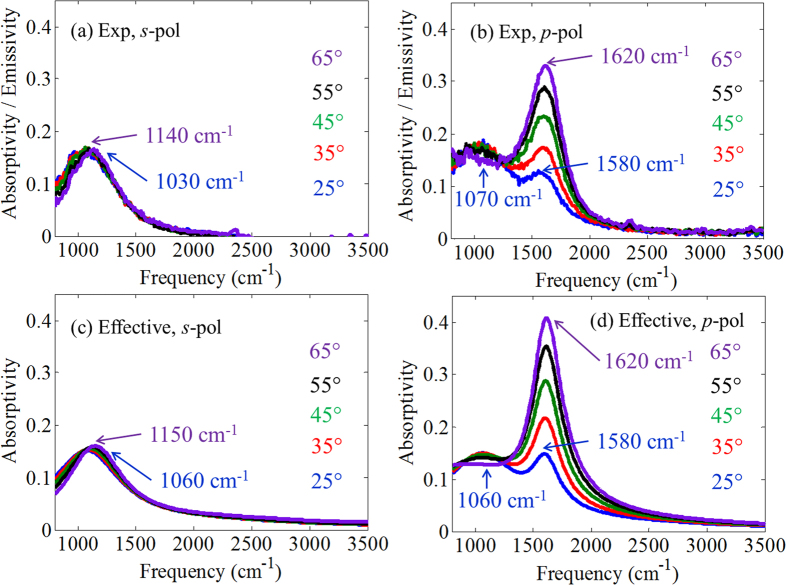
Experimental absorptivity/emissivity for (**a**) *s*-pol and (**b**) *p*-pol light. Theoretical absorptivity for (**c**) *s*-pol and (**d**) *p*-pol light. Trends are well predicted by the simulations.

The sample’s absorptivity/emissivity spectra were measured for both *s*-polarization and *p*-polarization at several angles of incidence using the ellipsometer (see the Methods section). (The experimental reflectivity and transmissivity spectra are reported in the Supplementary Material). Figure 3 shows a comparison of the experimentally measured absorptivity with the simulated data obtained using the effective medium model. The locations of the measured absorptivity peaks (~1060 cm^−1^ and ~1600 cm^−1^) are in good agreement with the simulations. Furthermore, the increase of absorptivity and blue shift of the ~1600 cm^−1^ peak with increasing angle observed in the experiment are well reproduced by the simulations. A similar blue shift with increasing incidence angle was also observed in ref. 35 for transmission. Simulations for the *p*-polarization absorptivity for varying number of pairs composing the SHM are reported in the Supplementary Material. We also report in the Supplementary Material an analysis of transmissivity versus frequency for two incidence angles and a Brewster characterization of our sample at frequencies in the type-I and type-II hyperbolic regions as well as in the elliptic region.

**Figure 4:**
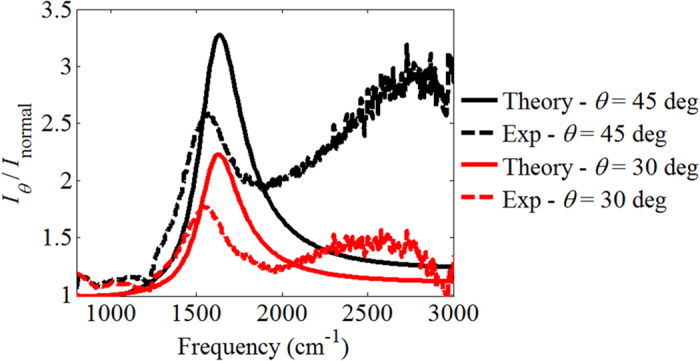
Experimental thermal emission ratio for unpolarized light. The theoretical result is computed for similar conditions. Trends are well predicted by the simulations.

Next we measured the sample’s thermal emission using a custom-built thermal emission measurement setup^36^. For these measurements, the sample mount was heated to a temperature of 300 °C. Only unpolarized spectra were obtained since the insertion of a polarizer introduces too much loss in our setup. We performed measurements at 0, 30, and 45 degrees incidence, with and without a sample present. Background measurements, obtained without the sample, were subtracted from the measurement obtained with sample in place. The emission spectra recorded at 30 and 45 degrees were normalized by the normal incidence spectra, and are plotted in Fig. 4 along with simulation results obtained using room temperature permittivities in the superlattice model. The peaks of the experimental emission are observed at ~1580 cm^−1^, in good qualitative agreement with the simulations (some spurious background is present at high frequencies). Thus the primary feature of the emission spectrum occurs close to the ENZ point of the doped quantum wells. The resonance frequency and directivity of the thermal emission are driven by an ENZ effect and the optical anisotropy of the hyperbolic metamaterial.

**Figure 5:**
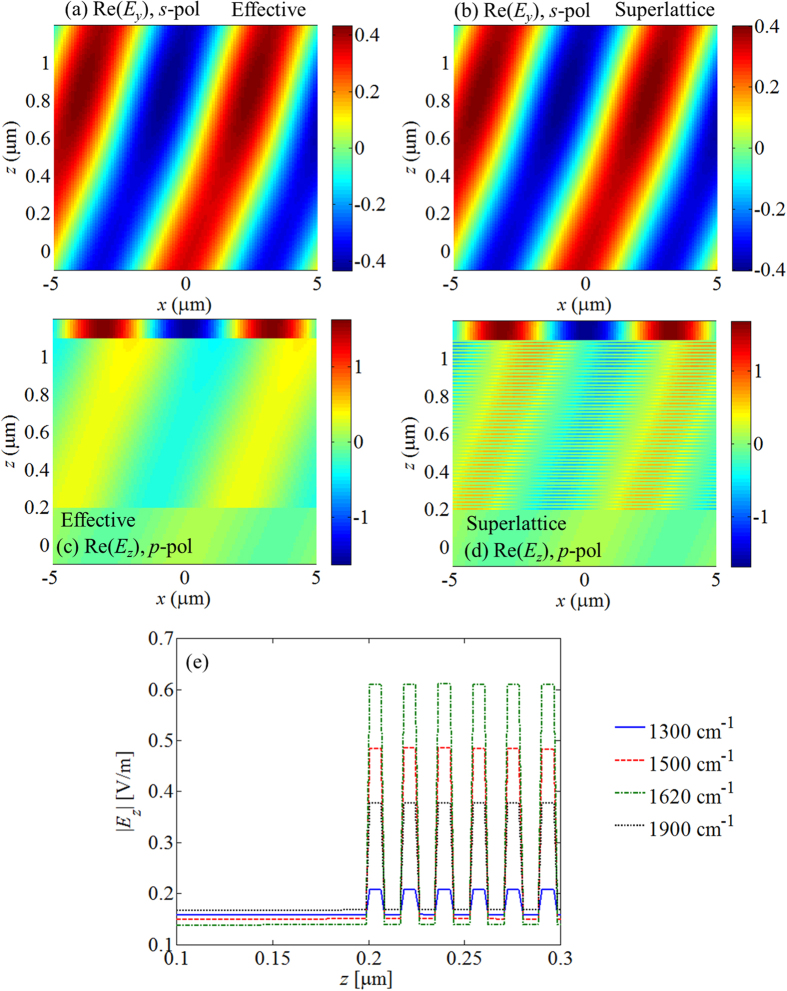
Field profiles under (**a,b**) *s*-pol and (**c,d**) *p*-pol incidence at 1620 cm^−1^ and 74 degrees, assuming the finite SHM structure in Fig. 1 with 50 layers. Results computed using (**a,c**) the effective medium approximation and (**b,d**) the superlattice implementation. (**e**) A line profile of the field at *x* = −2 μm across the first six doped layers. The fields are stronger at 1620 cm^−1^, leading to a stronger absorptivity in Fig. 2.

To further understand the origin of the thermal emission behavior, we investigated the electric field profiles within the SHM using both the superlattice model and the effective medium approximation. Figure 5 shows the field profiles corresponding to a frequency of 1620 cm^−1^ and incidence angle of 74 degrees. Note that this frequency is close to the epsilon-near-zero conditions of 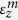 and 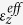. For the *s*-polarized field profile we plot the real part of the *y*-component of the electric field (Fig. 5(a,b)), while for the *p*-polarized profile we show the real part of the *z*-component of the electric field (Fig. 5(c,d)). Once again, we see a very good agreement between the effective medium and the superlattice models. However, for the *p*-polarized case, we see that the uniform electric field observed in the effective medium model (Fig. 5(c)) actually corresponds to a field concentration occurring in the doped quantum wells (Fig. 5(d)). Figure 5(e) shows a line profile (for *x* = −2 μm) of the real part of the *z*-component of the electric field for *p*-polarized incidence at 74 degrees for four different frequencies. Of these four frequencies, the largest fields occur at ~1620 cm^−1^, which is close to the frequency at which 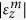 attains its smallest value of ~2.25 (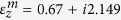). In this case, the continuity of the total displacement field normal to the surface suggests that the largest field should occur within the doped layer^20^,^37^ (provided the sample is not highly reflecting).

**Figure 6:**
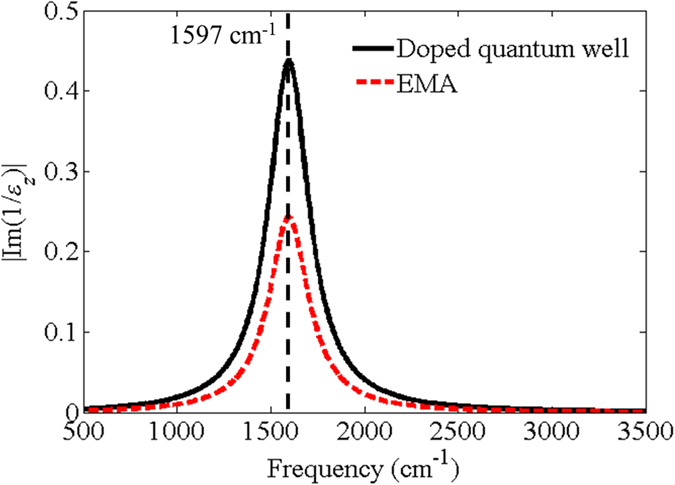
Magnitude of Im(1/*ε*_*z*_) versus frequency calculated using the measured permittivity of the doped quantum well and the effective medium model. Both models show peaks at 1597 cm^−1^.

To better understand the origin of the enhanced absorption/emission, we recall that absorption is proportional to the imaginary part of the permittivity and the magnitude squared of the field. A figure-of-merit that embeds these two quantities is 
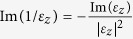
. Figure 6 shows 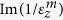 of the doped quantum well, along with 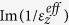 calculated using the effective medium model. A pronounced maximum is observed at 1597 cm^−1^ for both models, which is very close to the peak of the absorption/emission curves. Although this absorption peak does not occur precisely at the ENZ frequency (i.e. where Re(*ε*_z_) = 0), it is characterized by a small real part and large imaginary part of the permittivity which *arise directly from the ENZ resonance*. It is important to note that the correspondence of the peak absorption frequencies obtained with the two models is not a coincidence: inspection of the effective medium equations in the Methods section shows explicitly that 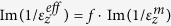, where *f* is the metal filling fraction. Thus, the spectral region of maximum absorption is solely dictated by the permittivity of the doped layer.

**Figure 7:**
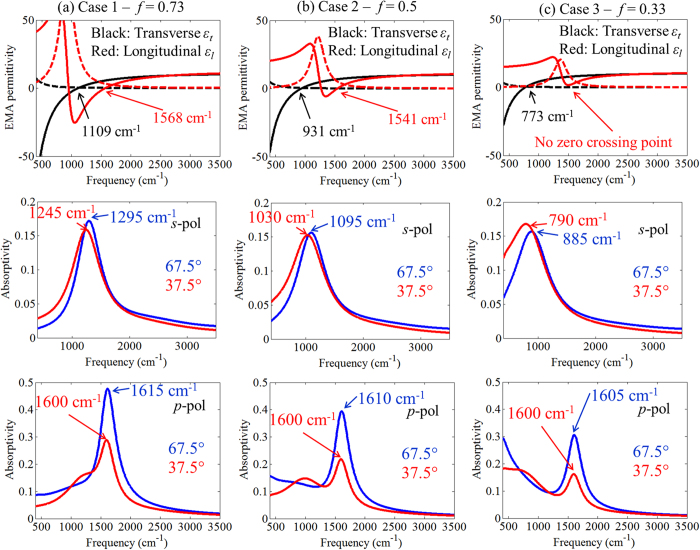
Effective permittivity functions of the homogeneous SHM (top row), and theoretical absorptivity for *s*-pol (central row) and *p*-pol (bottom row) light for three SHM cases: (**a**) 11 nm doped layer and 4 nm undoped layer, 60 periods; (**b**) 9 nm doped layer and 9 nm undoped layer, 50 periods; and (**c**) 5 nm doped layer and 10 nm undoped layer, 60 periods. The permittivity functions of doped and undoped layers are kept as in Fig. 1(b). In the top row panels, solid and dashed lines indicate real and imaginary parts of the permittivity, respectively.

To demonstrate the validity of our figure-of-merit, we numerically calculate the absorptivity for three different structures that are closely related to the structure shown in Fig. 1. The permittivities of the doped and undoped semiconductor layers are kept the same as those of Fig. 1, while the thicknesses of the layers are varied to vary the metal filling fraction (see Table 1). For each case, the number of layer pairs is modified to keep the same overall thickness of the SHM. The effective medium permittivities and polarized absorptivities corresponding to these cases are shown in Fig. 7. As seen in Fig. 7, a *p*-polarized absorption maximum is obtained near ~1600 cm^−1^ for all three cases, including Case 3 that does not support an ENZ condition of 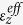, and 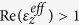. Since the underlying permittivities of the doped layers are the same in all cases, our figure-of-merit also predicts the peaks of 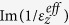 will occur at the same frequency for all cases. This confirms that the *p*-polarized absorption peak is associated with an epsilon-near-zero condition of the longitudinal component 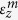 of the doped quantum wells, and supports our assertion that knowledge of the optical characteristics of the layers composing the SHM stack is necessary for a good understanding of the observed optical properties.

In conclusion, we have theoretically and experimentally analyzed the thermal radiation properties of semiconductor hyperbolic metamaterials. In contrast to the nearly flat and featureless mid-infrared thermal radiation spectrum observed in recent work on layered metal/dielectric hyperbolic metamaterials, we find strong directive and monochromatic emission features in proximity to the epsilon-near-zero frequency of the doped quantum wells of the SHM. We stress that this thermal emission behavior is obtained without the use of any periodic corrugation. Different thermal radiation properties are observed for *s-* and *p-* polarizations. Though all the thermal radiation characteristics are well recovered using an effective medium model for the SHM stack, their physical origin requires knowledge of the optical characteristics of the layers composing the SHM stack.

## Methods

### Anisotropic effective medium approximation

According to the local anisotropic effective medium approximation^34^, the sample in Fig. 1(a) can be described using a homogeneous, uniaxial permittivity tensor of the kind 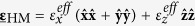, where


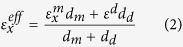


is the transverse permittivity along the transverse direction 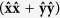 (parallel to the layers) and


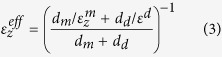


is the longitudinal permittivity along the longitudinal direction 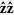 (perpendicular to the layers). In these expressions *d*_*d*_ and *d*_*m*_ represent respectively the thicknesses of the undoped and doped layers composing the SHM.

### Infrared variable angle spectroscopic ellipsometer

An ellipsometric measurement provides the quantity:


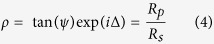


where *R*_*p*_ and *R*_*s*_ are the complex *p*- and *s*-polarized reflection coefficients. The measured *ψ* and Δ quantities are then fit to desired models using the WVASE software to extract the optical parameters of the models.

### Absorptivity and field calculations

Absorptivity and field calculations were performed using the transfer matrix method for the simulated layered structures. In the case of the superlattice implementation, we use a transfer matrix calculation with the permittivities derived from ellipsometry for each layer composing the superlattice. In the case of the effective medium approximation (EMA), we use a transfer matrix calculation with the effective permittivities using the anisotropic effective medium theory. We compute the reflectivity *R* and the transmissivity *T*, and then compute the absorptivity as *A* = 1 − *R* − *T*.

### Absorptivity measurements

We use the IR-VASE ellipsometer to measure angular reflectivity *R* and transmissivity *T*, and then compute the absorptivity as *A* = 1 − *R* − *T*.

## Additional Information

**How to cite this article**: Campione, S. *et al*. Directional and monochromatic thermal emitter from epsilon-near-zero conditions in semiconductor hyperbolic metamaterials. *Sci. Rep.* **6**, 34746; doi: 10.1038/srep34746 (2016).

## Supplementary Material

Supplementary Information

## Figures and Tables

**Table 1:** Parameters for Cases 1–3 differing from the superlattice in Fig. 1.

Case	Doped layer thickness [nm]	Undoped layer thickness [nm]	Metal filling fraction	Number of pairs
1	11	4	0.73	60
2	9	9	0.5	50
3	5	10	0.33	60
