# Supplementary material for: Directional and monochromatic thermal emitter from epsilon-near-zero conditions in semiconductor hyperbolic metamaterials
Source: Sci Rep. 2016 Oct 5;6:34746. doi: 10.1038/srep34746 (PMC5050433; doi:10.1038/srep34746)
Supplement: Supplementary Information [file srep34746-s1.doc]

**Supplementary material to “Directional and monochromatic thermal emitter from epsilon-near-zero conditions in semiconductor hyperbolic metamaterials”**

Salvatore Campione^1,2,*^ , Francois Marquier^3^, Jean-Paul Hugonin^3^, A. Robert Ellis^1^, John F. Klem^1^, Michael B. Sinclair^1^ , and Ting S. Luk^1,2,#^

^1^Sandia National Laboratories, P.O. Box 5800 Albuquerque NM 87185 USA

^2^Center for Integrated Nanotechnologies (CINT), Sandia National Laboratories, P.O. Box 5800 Albuquerque NM 87185 USA

3Laboratoire Charles Fabry, Institut d Optique, CNRS–Univ Paris-Sud, Campus Polytechnique, RD128, 91127 Palaiseau Cedex, France

[^*^sncampi@sandia.gov](mailto:*sncampi@sandia.gov)

[#](mailto:#tsluk@sandia.gov)tsluk@sandia.gov

This Supplementary material reports an analysis of the system with varying number of pairs composing the semiconductor hyperbolic metamaterial (SHM) and the experimental reflectivity and transmissivity of the sample. We also report an analysis of transmissivity versus frequency for two incidence angles and a Brewster characterization of our sample at frequencies in the type-I and type-II hyperbolic regions as well as in the elliptic region.

1. **Varying number of pairs composing the semiconductor hyperbolic metamaterial**

We report in Fig. S1 the absorptivity of the SHM composed with 25, 50, and 75 pairs computed via the superlattice model. One can notice that while the high absorption close to ~1600 cm^-1^ is present in all the three configurations, the absorption around ~1060 cm^-1^ can be engineered.

Fig. S1. Theoretical absorptivity for *p*-pol for a SHM with (a) 25, (b) 50, and (c) 75 pairs computed using the superlattice model.

1. **Experimental reflectivity and transmissivity of the SHM sample**

We report in Fig. S2 the experimental transmissivity and reflectivity of the SHM sample in the manuscript.

Fig. S2. (a, b) Experimental transmissivity and (c, d) reflectivity of the sample.

1. **Transmissivity versus frequency**

We report in Fig. S3 the theoretical transmissivity (for *s*- and *p*-polarization) of the SHM sample in the manuscript versus frequency for two angles of incidence computed using the superlattice model: at 0 degrees and at 45 degrees. One can note a low transmissivity in the type-II hyperbolic region, and a high transmissivity in the type-I hyperbolic region.

Fig. S3. Theoretical transmissivity of the sample for (a) *s*-polarization and (b) *p*-polarization versus frequency for two angles of incidence computed using the superlattice model.

1. **Brewster angle characterization**

We report in Fig. S4 the theoretical reflectivity (for *s*- and *p*-polarization) of the SHM sample in the manuscript versus angle of incidence for three frequencies computed using the superlattice model: at 800 cm^-1^ in the type-II hyperbolic region; at 1300 cm^-1^ in the type-I hyperbolic region; and at 2000 cm^-1^ in the elliptic region. One can note that a Brewster angle (i.e. zero reflectivity) is not observed for *s*-polarization. For *p*-polarization, a clear Brewster angle is achieved in the elliptic region; a pseudo-Brewster angle is observed in the type-I hyperbolic region; almost no Brewster angle is observed in the type-II hyperbolic region.

Fig. S4. Theoretical reflectivity of the sample for (a) *s*-polarization and (b) *p*-polarization versus angle of incidence for three frequencies computed using the superlattice model.
